# The trinomial health, safety and happiness promote rural tourism

**DOI:** 10.1186/s12889-023-15849-8

**Published:** 2023-06-19

**Authors:** Rafael Robina-Ramírez, Rafael Ravina-Ripoll, Francisco Javier Castellano-Álvarez

**Affiliations:** 1 Department of Business and Sociology, University of Extremadura, Cáceres, Spain; 2 University of Cádiz, Cádiz, Spain; 3 Department of Economic, University of Extremadura, Cáceres, Spain

**Keywords:** Health and safety protocols, Happiness, Managers, Rural tourism, Rural areas, Tourist resources

## Abstract

**Background:**

Health and safety protocols have become a requirement to promote rural tourism (PRT). From this perspective, this paper empirically analyses how the health and safety dimensions influence the happiness of hotel managers and rural tourists in the post-Covid 19 era.

**Methods:**

A theory-based structural equation model will be carried out of activation of norms, that measure variables: sanitary, socioeconomic, and safety. Precisely, we will measure how those three attributes affect the managers-guests’ health in rural areas and their search for happiness at the rural destination. Based on the above, a field of study has been 215 rural tourist accommodations in the Extremadura region (Spain) and a sample population of 443 guests. Data were organised through the SEM-PLS path modelling.

**Results:**

The results achieved statistically show the need to undertake a new model of healthier and safer tourism consumption that values the tourist resources of rural areas, especially nearby and sustainable destinations, based on the guiding principles of safety, health, and happiness.

**Conclusions:**

The first conclusion is that promoting tourist destinations under safe and healthy conditions has become a priority objective in the tourism industry. The second conclusion that follows from the first is that the variables safety and health and the pursuit of happiness are essential factors in promoting tourist destinations for rural hotel managers and rural tourists. The third conclusion related to the first two is that the opportunity that this study provides to develop strategies of an innovative, sustainable, and creative nature based on the relationships of the new trinomial of health, safety and happiness, from the perspective of happiness management.

## Introduction

Recently, the World Health Organization (WHO) has published monographs on health and safety procedures. Many of them refer to topics related to the safety of clinical procedures, HIV prevention protocols, intensive care units, hand-washing techniques, etc [1]. Under this umbrella, the pandemic caused by Covid-19 led public administrations to double exponentially the creation of preventive health actions aimed at drastically reducing the spread of this virus [2]. In this way, governments seek to guarantee the physical well-being of citizens at the expense of the progress of economies. This assertion is motivated by the fact that many of these recommendations minimise the productive and tourism development of the territories [1].

In the same vein, to ensure the welfare of citizens, the European Commission has established a series of recommendations to ensure the safety of free movement in the European Union [3]. In Spain, regulations aimed at the gradual opening of bars, restaurants and hotels were approved under the guiding principles of safety, health, and confidence [4]. Examples of these measures include social distancing [5], physical distancing, quarantine and isolation, measures or technological systems to track the virus in people [6]. Such protocols have provided greater security for both tourism businesses and visitors coming to Spain and a greater degree of information to predict health deficiencies and make timely decisions [7].

Therefore, promoting tourist destinations under safe and healthy conditions has become a priority objective in the tourism industry [8]. Ensuring both factors allow tourism consumers to enjoy great positive experiences [9]and to lead healthy habits during their stay in tourist accommodations, both aimed at developing happiness [10, 11]. Given the above, the promotion of destinations leads tourism managers to cultivate a management model based on the theories of happiness management and the search for the subjective well-being of their users through the parameters of quality, health, and safety [12].

From this perspective, this article has a double academic purpose. The first one is on how health and safety variables and the pursuit of happiness are essential factors in promoting tourist destinations for rural hotel managers and rural tourists. Moreover, the second, to fill the existing gap in the literature to demonstrate that the new trinomial of health, safety and happiness constitute key vectors to convey strategies of an innovative, sustainable, and creative nature that act as a driving force for tourism activity in the post-Covid-19 era. Hence, the need to implement public policies that promote the well-being of tourists through the promotion of exciting and healthy activities for people [13].

After this introduction, the following section presents the conceptual framework. The third section develops the methodology used and describes the structural equation technique used and the dimensions of our theoretical model. The fourth section presents the results obtained in this academic work. Moreover, the last section formally shows the discussions and conclusions of the study, as well as future lines of research.

## Theoretical framework

Based on what has been read so far, this scientific study aims to statistically illustrate the potential of the dimensions of safety and health and their effect on happiness. The study will analyse whether these variables become dynamic and innovative forces that promote tourism destinations in rural ecosystems according to the guiding principles of excellence, well-being, quality, and respect for the environment. In the following sub-sections, a literature review of all the variables that make up the academic corpus of this article will be carried out. It will serve to establish the theoretical corpus of our research hypotheses.

### The promotion of rural tourism (PRT)

In the second quarter of 2020, most leading countries in the tourism sector ceased promotion and marketing activities in response to travel restrictions [14–16]. During the early months of the pandemic, promotional strategies were replaced by planning for the uncertain future of businesses and raising the financial support needed to stay in business [17].

In the new Covid-19 era, the promotion of destinations must consider the terrible effects of the pandemic on both the demand and supply side of tourism product sales [18]. On the demand side, this crisis will bring a reduction in disposable income with a consequent reduction in tourists’ willingness to pay [19]. On the supply side, the innumerable restrictions on mobility and closures of companies in the sector have caused total or partial closure during specific periods changing the consumption patterns of tourists and, consequently, the commercial strategy of supply.

These new consumption patterns lead us to stop and explore the role played by the two essential variables that guarantee the tourist experience: safety and health, and how both affect the tourist’s happiness.

### Safety (S) as the axis of healthy tourism

This sudden onset of a pandemic crisis is pushing employees into current stressful situations in their workplace [20, 21], eroding trust between employees and managers and sometimes ending up affecting workers’ mental health and living conditions [22]. These adverse social and organisational working conditions can turn the workplace into a toxic place not free of extreme pressures, leading to states of fear, paranoia, and anxiety [23].

In the current context of the pandemic, health authorities have proposed that tourism businesses need to ensure the safety of both the service provider and their customers [1]. This new approach to experiences is based on two dimensions; “Ad intra”, adapting the new meaning of “safety” to those who provide the experience, and “ad extra”, generating sufficient confidence in customers. Thus, the service must comply with the characteristics of being hygienic, social distancing, and safety protocols established by the country’s health authority. This double characteristic defines the “safe” experience.

According to Anderson et al. [24], not only is it important to comply with sanitation protocols, but it is also necessary to have the individual cooperation of both customers and employees in order to provide a service without the risk of contagion. This is crucial to reduce the spread of COVID-19. This collaborative process begins when Non-Pharmaceutical Interventions (NPIs) known as individuals take responsible actions in their daily lives that go beyond vaccinations and medication. These actions include isolating themselves at the first perceived severe symptoms, and generally taking care of social distancing [25].

These responsible measures to reduce infection through Non-Pharmaceutical Interventions (NPI) are not only necessary to promote personal hygiene. It provides tourists with a degree of confidence of a psychological nature to overcome the brakes that may be put on any travel to a tourist destination. When responsible measures are employed to reduce infections, it helps tourists to overcome psychological barriers by providing an added motivational factor for travel [26].

These psychological and social components based on providing safety to tourists allow the deployment of Non-Pharmaceutical Interventions (NPI) to depend not only on individual responsibility measures but also on community behaviour. Group action is even more important than individual actions to make correct and responsible use of NPIs to ensure safety [27].

According to Huang et al. [28] attention to these psychological factors falls within the theory of planned behaviour and health belief model. Among this psychological health, factors would be perceived susceptibility, perceived severity of the situation, and perceived benefit of taking protective health measures. Awareness in favour of these three factors leads to health behaviour that helps to prevent any kind of risk. Although the knowledge of the seriousness of the situation and the tourists’ susceptibility to it may slow down the preparation for the trip due to the fear of becoming infected, the mere interest in implementing protective health actions increases the motivation to undertake the trip. These health actions help to overcome the possible negative emotions arising from the uncertainty of the possible spread of the virus. Responsible exercise of health actions increases psychological resilience against the fear of not travelling [28].

Psychological resilience depends on individuals’ level of risk tolerance. Tourists who have a high degree of rationality in assessing all the consequences of travel under pandemic conditions may reduce their fear of contagion when such health measures are employed [29]. Psychological resilience may vary depending on the type of information available about travel, the procedures employed to reduce risk, and individual and community actions to protect against the virus [29]. Psychological resilience increases when the effects of NPIs are shown to be successful. Thus changes in tourist behaviour as a result of COVID-19 control measures lead to increased satisfaction among tourism employees [29].

Tourism has so far been understood as the primary producer of consumer experiences and expectations [30]. The term “safety” has traditionally been applied to adventure tourism and the safety measures taken to avoid any kind of risk [31]. Risks identified include physical, financial, equipment or functional and health risks. Other types of risk associated with tourism are the risk of political instability due to political turmoil in the destination country even up to the level of a terrorist attack, the risk of dissatisfaction from a travel experience that has not met tourists’ expectations, and social risk when social activities experienced during the trip may give rise to criticism. To overcome these risks, it is possible to take measures beyond those suggested by public authorities. When the risks of contagion from pandemic situations are combined with other types of risks mentioned above, it is necessary to analyse the risk tolerance level of each individual tourist to avoid turning the tourist experience into an unfortunate one. In order to do so, it is necessary to make a prior effort to assess the consequences that such a trip may have on the tourist [32]. When the potential consequences exceed the level of risk tolerance, the tourist should abandon the travel project and switch to a destination with a lower level of risk exposure.

Several methodologies exist to reduce the level of risk exposure, including seeking up-to-date information from the destination on how to avoid or minimise perceived risk and incorporating measures that adjust tourist behaviour to minimise its impact or avoid a risk [33].

These two methodologies can be accompanied by the optimism-pessimism theory proposed by De Jonge, et al., (2008) [34]. or the Theory of Protective Motivation (TMP) of Floyd et al. (2000) [35]. The former focuses on defining the aspects that influence the tourist’s perception of security depending on the tourism product or service he/she chooses. If the choice is based on the acceptance of a tourist product or service with hardly any risk, we speak of a safe or optimistic scenario. If, on the other hand, the tourist perceives some assumption of risk in the model of the product or service he/she contracts, we speak of a pessimistic scenario. Both scenarios can occur in the same destination when activities or experiences are combined that may end in circumstances that entail greater or lesser implicit risk.

Protective Motivation Theory (PMT) allows the tourist to cope or not with environmental or personal threats when preparing for a trip that involves some kind of health risk [35]. The first is known as “adaptive coping” towards an unsafe destination when all personal, social and organisational means are employed to resolve the risk before embarking on the trip. The second is ‘maladaptive coping’ when there is an unwillingness on the part of the tourist to resolve the safety risk situation, when the situation is well beyond the tolerance threshold, or the risk is too high [36].

Whereas in adaptive coping there is a commitment to use all means to protect the health, in maladaptive coping decisions are made without consideration of the use of protective measures [35, 36].

However, with the emergence of the pandemic, the concept of “safety” has shifted from adventure tourism to the health connotations of tourism. In hotels, the model of designing empathetic and unique experiences has shifted to contagion-free tourism experiences [9].

Governments alone cannot address this challenge. Hence protocols are designed to reduce uncertainty and stress, convey safety, and ensure healthy modes of operation [37]. In this context, tourism businesses must develop a complimentary planning and awareness effort to develop safe experiences [38] through social distancing and limiting people-to-people contact to reduce viral transmission [39]. The tourism industry is at high risk of infection among workers and tourists, and therefore decisions need to be made, such as canceling group meetings; holding meetings virtually; keeping children away from group settings; and promoting online connections between people or social networks to reduce the spread of Covid-19 [40].

Regarding social distancing, the implementation of sanitation and hygiene measures is essential for hotels. WHO has provided practical guidance on providing safe water, sanitation, and hygienic conditions since the origin of the spread of the Covid-19 virus [41]. Rural areas are exposed to worse sanitation services to meet their basic social and health care needs [42]. Rural/urban inequities, combined with inequities within rural areas in health, health care and financial resources, cause particular challenges for Covid-19 health and well-being [43]. In the case of Spain, these guidelines have been complemented by a series of measures to prevent infections and contagions in hotels [44]. Along with social distancing measures and sanitary and hygienic measures, the health authority in some regions of Spain has promoted the distribution of Covid-19 tests throughout the country to curb contagious diseases [4].

Based on these cognitive arguments, the following hypotheses are formulated:

#### Hypothesis 1

Safety seeking in rural tourism destinations (S) influences health-seeking in rural tourism destinations (H.T.).

#### Hypothesis 2

The search for safety in the rural tourism destinations (S) influences the happiness sought by the tourist in a rural tourism destination (H.P.).

#### Hypothesis 3

The search for safety in the rural tourism destination (S) influences the promotion of the rural tourism destination (P.R.T).

### Health (H.T.) in the rural tourism sector

Having analysed the factors that define the safety of a tourist destination, we now turn to health as an attribute that has been especially valued due to the pandemic caused by Covid-19. Even though COVID-19 has had a major impact on the tourism industry globallyit has been less affected in rural areas [45]. One region that has seen an increase in rural tourism during Covid 19 time in Extremadura, a region in western Spain known for its natural beauty, rich history, and rural way of life [46, 47]. Rural tourism in Extremadura has increased during the COVID-19 pandemic [48]. Many people living in large cities and crowded tourist destinations are searching for quieter, more remote locations. Extremadura’s rural areas offer just that, with plenty of space and fresh air, making them an attractive option for travelers looking for a break from the hustle and bustle of city life. Furthermore, Extremadura’s rural areas offer a wide range of activities for travelers, such as hiking, birdwatching, and cultural tours. Extremadura is a popular destination for outdoor enthusiasts. The region is also home to numerous natural parks, such as Monfragüe National Park and Sierra de Gata, where visitors can experience the beauty of the region’s wildlife and flora [49].

Those reasons make Extremadura popular among health tourists, as they offer a peaceful and natural environment for healing and rejuvenation. Rural tourism offers a quieter and more relaxed experience compared to busy urban areas, making it an attractive option for those seeking to escape the stresses of everyday life and avoid crowded places. On the other hand, social distancing measures and health concerns have also led many travelers to seek accommodation in rural homes or nature areas where safe distances can be maintained, and crowds avoided [50].

There are multiple meanings of the concept of health tourism [51] and its discipline, wellness tourism. It offers tourists a wide range of infrastructures and healthy activities to significantly boost their hedonic and psychological happiness [52]. One of the basic premises for achieving this goal is the natural resources offered by rural ecosystems. These spaces open the door to positive experiences and the emotional well-being of their visitors [53]. In this way, rural tourism becomes a vital health catalyst for tourists to directly contact nature and the environment [54].

The interests of these studies lie in demonstrating that happy human beings have high chance of enjoying a healthy, whole, and long life [55, 56]. This fact is motivated, among other factors, because these individuals suffer less stress or illness and seek their well-being basically through family, sociability, sport, religion, or tourism [57, 58].

Regarding this last variable, it is worth noting that there is extensive scientific literature on tourism management theories today. Many argue that leisure travel is a source of health and positive experiences [59]. Both of these elements, along with many others, possess an important influence on the happiness of citizens, especially in times of economic crisis such as the one we are experiencing in the current post-Covid-19 era [60]. This cognitive reasoning makes it unsurprising that wellness and health tourism has registered high growth rates internationally over the last few years [61]. Behind these figures is that one of the main eudaimonic motivational factors people have to choose a tourist destination is health. A resource that provides tranquillity that relaxation, and subjective well-being [62].

According to this theoretical substantiality, the intangible resource of happiness stands as a new Keynesian variable of wellness tourism from the perspective of happiness management [63]. Perhaps, this justifies the appearance of a limited number of investigations showing inferentially that tourists’ hedonic and eudaimonic happiness decreases significantly after holidays [64]. Such a finding shows that leisure travel undertaken by people with the idea of vivifying their state of health has very positive short-term effects on their life satisfaction. In contrast to this result, other types of work on the topic of tourists’ experiences are based on examining the satisfaction that people enjoy during their stay as tourists [65]. In this sense, it should be noted that many of these scientific works are characterised by reaching very different theoretical and practical conclusions [66].

As a result of this research, there has been an interesting academic debate in recent years on the psychosocial determinants that tourism generates on the dimensions of health and happiness for both residents and visitors [67]. Some of these studies suggest that the empirical analysis of both constructs should be further deepened to have solid literature on the impact of these two parameters on health tourism [68, 69]. Based on these previous arguments, a significant number of authors argue that health tourism management models should pivot on the holistic search for the happiness of tourists under the trinomial infrastructure, destination, and territory [70].

Many scientific studies are currently adding to this line of research in two main ways. The first is exploring the destination’s promotional offers as a robust boost of health tourism in rural areas [71]. An issue not considered by both public administrations and tourism industry professionals. Moreover, the second is that happiness is crucial in boosting health tourism in rural areas sustainably and competitively [72].

All these cognitive arguments lead us to propose the following research hypotheses:

#### Hypothesis 4

Health seeking in rural tourism destinations (H.T.) influences rural tourism destination promotion (P.R.T).

#### Hypothesis 5

Health seeking in rural tourism destinations (H.T.) influences the happiness seeking tourists in rural tourism destinations (H.P.).

### Happiness in the rural tourism sector (HP)

Having analysed the effects of safety and health on happiness, we now study the influence of happiness as an essential factor when promoting a tourist destination.

Happiness is understood as the valuation of an internal mood or emotional state that allows the individual to define those aspects or conditions of life that best suit their way of thinking and deciding [73]. Happiness, as a universal human desire, plays a fundamental role in the promotion of tourism destinations [74]. When people travel, they tend to seek experiences that make them happy, and tourism organisations can capitalise on this by promoting destinations known to foster positive emotions and well-being [75]. As the tourism demand is growing every year [76], not only economic determinants have been the consequences of that growth.

According to [77] from data extracted from the World Happiness Report, which is composed of an index that collects not only the degree of happiness of the inhabitants of the country but also the degree of happiness that visitors reach in those countries, we observe how the happiness variable becomes another factor to promote tourist destinations.

McCabe and Johnson [78] argue that tourism contributes positively to the happiness of individuals, provided that there is a tourism model that stimulates the subjective well-being not only of visitors but also of its residents [79–81].

The tourism model depends on the conditions of a destination based on the level of services provided, the economy of the territory, cultural identity, and care for the environment. All these variables influence tourism promotion and tourist happiness. In addition to this, the tourist’s perception and formation are the results of the different aspirations, emotions, and expectations of the tourist [75]. Together with the type of tourism offered by a destination and the level of aspirations of the visitor, we observe another variable that influences the happiness of the tourist and the promotion of a destination.

According to Doran et al., (2015) [82] the theory of social comparison influences the level of happiness of the individual. This comparison arises when individual happiness is contrasted with other successful life outcomes such as superior health, higher income, and closer social relationships with work or community members [83]. However, tourists not only measure the degree of happiness they obtain on an individual level but also relate it to the destination, and this contrast can influence its development and promotion [84]. Empirical findings suggest that well-being in terms of the pursuit of happiness is strongly correlated with comparative analyses either with the same person or with other people in different destinations [83].

The role that happiness plays in the promotion of a tourist destination is also related to the theory of tourist attraction [85]. An experience of happiness in a tourist destination leads to attracting new tourists. These experiences of happiness have an impact on the residents who support tourism development which shapes a tourist’s memorable experience and influences their likelihood to return to the destination [86]. Nawijn and Mitas (2012) [87] and Andereckand Nyaupane (2011) [88]. It is therefore essential that a destination actively seeks the well-being of its residents and, in doing so, enhances their happiness.

In this sense, Ivlevs [89] highlights that the actions developed by tourists during their holidays dampen the happiness of the residents of tourist destinations. In contrast to these theories, recent studies show no economic and sustainable progress in the territories where tourism occurs [90]. Rural tourism is not exempt from this reality [91]; spaces are characterised because visitors’ happiness cannot be separated from the residents in tourist destinations. It should not be forgotten that both actors are consumers of leisure activities that improve their life satisfaction, such as restaurants, accommodation, or historical heritage [22]. From this conjunction of interests of subjective well-being, new academic studies consider that the choice of rural tourist destinations is often predetermined by the happiness offered by the landscape where people want to enjoy their holidays [92] being aware that such a decision cannot be separated from other psychological and microeconomic elements that also contribute to the happiness of tourists [88]. The cross-cutting concept of happiness is determined by the dimensions of materiality, safety, expectations, nature, or health [93, 94].

In short, happiness becomes an attractive intangible resource that helps, on the one hand, to make individuals loyal to specific tourist destinations, and on the other hand, to design modern tourism marketing strategies aimed at showing those rural tourist destinations are sources of positive emotions, satisfaction, and health [95]. In sum, with all the above, the first research hypothesis of this paper is put forward:

#### Hypothesis 6

visitor happiness (S) influences the promotion of rural tourism destinations (PDT).

## Methodology

### The behavioural transformation model based on the Schwartz model

According to Schwartz [96] behavioural transformation begins from the knowledge of the consequences of an action (consequence awareness: C.A.) which generates responsible behaviours (assignment of responsibility (A.R.) and personal obligations to action (personal norms (P.N.).

Translating the Schwartz theory into the paper topic is worth saying that the consequences of a pandemic (consequence awareness: C.A.) lead to responsible actions through the development of responsible behaviours (R.B.). This behaviour is based on exploring the role played by “safety” and “health” by hotel managers and customers. Hence, responsible behaviour generates behaviour-transforming norms (T.N.) in two directions: “Ad intra”, adapting the new meaning of “safety” to those who provide the experience by complying with the characteristics of being hygienic, social distancing, and safety protocols established by the country’s health authority and treating “health” as a discipline related to wellness tourism offers tourists (Schwartz and Howard [97].It provides a wide range of infrastructures and healthy activities to be adapted to safety conditions. “Safety” and “Health” affect the tourist’s happiness by providing hedonic and psychological happiness that stimulates the subjective well-being not only of visitors but also of its residents.

### Selection of indicators and model

According to Sánchez-Oro Sánchez and Robina-Ramirez [98], for selecting indicators, it is necessary to use a methodology that allows contrasting the information obtained from the literature review with the sample of the population to which the study is addressed. In this sense, group interviews have been used through focus groups to collect qualitative data. These data have allowed us to design the variables that feed the statistical model used.

For this purpose, a group of hotel managers from each region in Spain was randomly invited to participate in two focus group sessions. Only 32 responded favourably to participate in two focus group sessions. During the fourth week of June 2021, two focus groups were conducted through the online platform zoom to select the indicators for each latent variable extracted from the literature review.

Each of them was sent emails explaining the scientific proposal of the research, as well as the methodology followed, which is based on two steps. In the first step, the meaning of health, happiness, and safety in the context of the management of rural hotels during the pandemic is explained in detail. During approximately one and a half hours, the research team could contrast the literature review with the feedback received on developing questions to implement the questionnaire. During the second meeting, the meaning of the indicators was reviewed and modified. The results are shown in Table 1.

**Table 1:** Preliminary study and list of items corrected by the managers

Indicators			
PRT: Promotion of Rural Tourism			
**(PRT1)**	The promotion of rural tourism is based on the search for the happiness of tourists and residents.		Kwon & Lee (2020); Bimonte & Faralla (2016); Pratt et al. (2016)
**(PRT2)**	Promotion of rural tourism based on the health of tourists and residents.		Cini et al. (2013)
**(PRT3)**	Promotion of rural tourism based on security		WHO (2020,2021)
H.P.: (Happiness) The quest for health in the rural tourism destination.			
**(HP1)**	Considers that happiness is a source of pleasure that contributes to the enjoyment of well-being, to a peaceful and self-sufficient life.		Khalil (2019); Kwon & Lee (2020); Bimonte & Faralla (2016); Pratt et al. (2016)
**(HP2)**	In addition to subjective well-being, satisfaction or quality of life, he believes that happiness is found in the personal growth of individuals.		Dhiman (2021)
**(HP3)**	Happiness contributes to the economic and sustainable progress of the territories where tourist activity takes place.		Seresinhe et al. (2019)
**(HP4)**	Happiness helps to build the loyalty of individuals to tourist destinations and to design tourism marketing strategies.		Chen & Li (2018)
H.T.: (Health) The quest for health in the rural tourist destination			
**(HT1)**	Travelling for pleasure is a wellspring of health and positive experiences.		Burns & Crisp (2021)
**(HT2)**	Health tourism is closely related to hedonic (pleasurable) and psychological happiness.		Hritz et al. (2014); Akhoundogli & Buckley (2021)
**(HT3)**	Rural ecosystems allow you to enjoy experiences of health and emotional well-being in contact with nature and the environment.		Hall (2010); Puczkó & Smith (2012)
S: (Security) The search for security in the rural tourist destination.			
**(S1)**	Under the effects of the pandemic, tourists seek contagion-free tourism experiences.		Robina-Ramírez et al. ( 2021b,a)
**(S2)**	Tourism companies have to guarantee the safety of the service provider and their customers.		WHO (2020,2021)
**(S3)**	Tourism businesses must develop a complimentary planning and awareness effort to develop safe experiences.		Moreno-Luna et al. (2021)

Own source

**Fig. 1:**
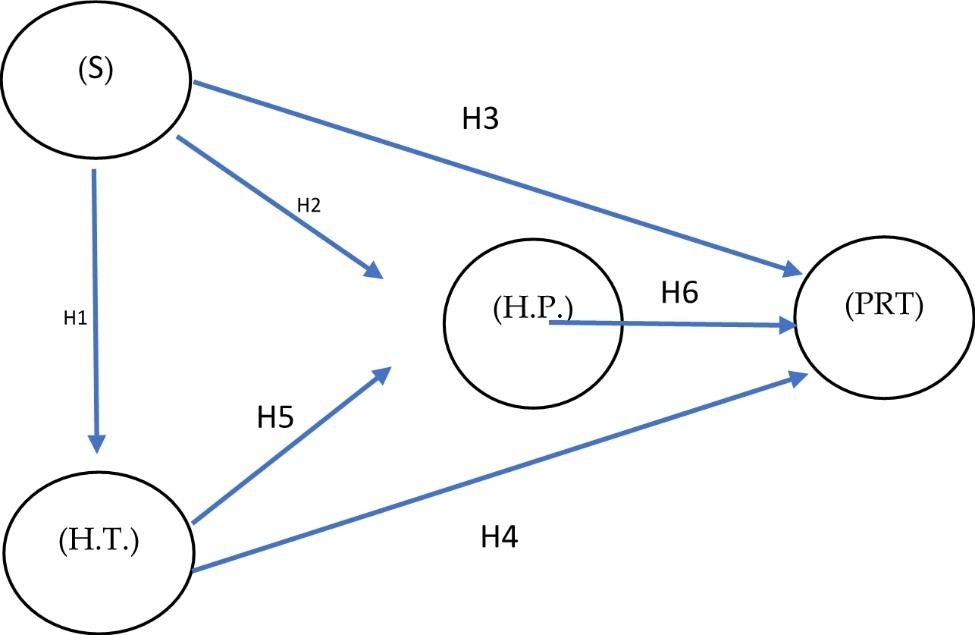
Presentation of the model Source: Self-made

The variables extracted from the literature review are 4: (1) PRT (Rural Hotel Promotion), (2) H.P. (Happiness): The pursuit of happiness in rural tourism destination, (3) H.T.: (Health) The pursuit of health in rural tourism destination, (4) S: (Security) The pursuit of security in rural tourism destination. The model presented is shown in Fig. 1:

### Population and sample

At the beginning of the research design, the regional government tourism offices of the 17 communities that make up Spain were contacted. From this meeting, we were provided with the addresses of the rural tourism hotels that had survived the first year of the Covid-19 era.

During the first week of June 2021, the authors of this article contacted the director of each entity by telephone after sending a letter of introduction. The letter explained the scientific objective of the study and the desire to know the importance of measuring the impact of health, safety, and happiness in the promotion of rural tourism.

Throughout June, the research team received emails from rural hotel managers. During July and August, the responses to the emails were received by sending a link that opened a questionnaire form in google-doc; the participation reached 232 participants, of which 17 were erroneous. The final sample was 215 hotel managers (Table 2).

**Table 2:** Population and sample

Spanish Regions	Urban hotels	Sample	Rural hotels	Sample
Andalucía	1147	70	2037	77
Aragón	429	29	822	24
Asturias, Principado de	134	14	227	11
Balears, Islas	63	13	122	10
Canarias	286	15	514	18
Cantabria	93	10	267	12
Castilla - La Mancha	543	18	1452	61
Castilla y León	707	42	2717	81
Cataluña	988	55	1576	32
Comunidad Valenciana	676	42	675	16
Extremadura	272	19	573	21
Galicia	891	54	296	20
Madrid, Comunidad de	727	41	222	10
Murcia, Region de	98	12	139	13
Navarra, Comunidad Foral de	173	14	725	15
País Vasco	354	21	323	13
Rioja, La	89	6	91	9
Total	7670	475	12,778	443

Own source

Regarding the hotel managers, most of the participants were men (63%), with ages between 30 and 39 years (32%) and 40–49 (27%) mainly (Table 3). Regarding the level of education, 70% had university studies and master’s degrees. It shows the high level of preparation of those responsible for tourism establishments in rural areas.

**Table 3:** Sample composition

	Hotel Managers		Tourists	
Gender	N = 215	%	N = 443	%
Female	78	0,36	250	0,57
Male	136	0,63	185	0,42
No respond	1	0,00	2	0,00
Total	215	1,00	437	1,00
Age	N = 215	%	N = 443	%
18–29	32	0,15	145	0,33
30–39	69	0,32	142	0,32
40–49	58	0,27	96	0,22
50–59	45	0,21	42	0,09
More than 60	9	0,04	15	0,03
No respond	2	0,01	3	0,01
Total	215	1,00	443	1,00
Level of studies	N = 215	%	N = 443	%
Primary Education	7	0,03	22	0,05
Secondary Education	11	0,05	41	0,09
Bachelor	43	0,20	153	0,35
University	133	0,62	175	0,40
Master	18	0,08	48	0,11
No respond	3	0,01	4	0,01
Total	215	1,00	443	1,00

About the tourists who participated in this study, a higher percentage of women (57%) have stayed overnight in rural hotels during July and August. Most of the tourists were young and middle-aged. 87% of the tourists were under 49 years old. Compared to the profile of the hotel managers, the educational level of the tourists is lower; 51% have reached a university or master’s degree level. However, the percentage of tourists with a bachelor’s degree (35%) is higher than that of hotel managers (20%).

### Tabulation of data from PLS-SEM

PLS-SEM is an experimental methodology based on primary or secondary data. This methodology is well suited for making predictions. It does not require a normal data distribution and is adaptable to small sample sizes [99]. PLS-SEM indicates the importance of relationships between constructs and can handle numerous independent variables simultaneously [100]. Bootstrapping-based methods are used to assess the overall model fit in PLS, which seems to work quite well, as indicated by [101]. This statistical technique is observed when establishing dependency relationships between latent variables and indicators [102].

The PLS (Partial Least Squares) technique SmartPLS 3 Version 26 was applied to build the statistical model. This version is especially recommended for composite site models [103]. The SEM-PLS method allowed the development of two models: the measurement model and the structural model. To proceed with the structural model analysis, the authors of this paper analysed the reliability between indicators and constructs and the validity of the measurement model [79.100]. In this case, reflective items were used because they are interchangeable [104].

## Results

### Data analysis and measurement model

The data have been processed using the multivariate PLS technique. This approach is recommended for social science analysis [105] in small samples [106]. According to [107], individual reliability should be analysed from the beginning of the methodological process of determining the appropriate indicators. In this sense, the loadings (λ) should be greater than 0.707 (Table 4).

**Table 4:** External model loads

	Hotel managers				Rural touristic			
	HP	HT	PRINT	S	HP	HT	PRINT	S
HP1	0,864				-------			
HP2	0,831				0,911			
HP3	-------				0,903			
HP4	0,842				-------			
HT1		0,792				-------		
HT2		0,826				0,907		
HT3		0,802				0,891		
PRT1			0,818				0,892	
PRT2			0,896				0,777	
PRT3			0,902				0,895	
S1				0,725				0,928
S2				0,788				0,926
S3				0,770				-------

Own source

Table 5 examines the individual reliability of Cronbach’s alpha [108] and Rho_A with values > 0.70 [80, 101] and composite reliability with values > 0.5 [84, 106]. The minimum level of average variance extracted (AVE) explains that all the analysed constructs were reliable, with more than 50% variance between their indicators [109]. In our case, all constructs exceed the minimum values of composite reliability and convergent validity.

**Table 5:** Reliability, validity

	Hotel managers				Rural touristic			
	Cronbach Alpha	rho_A	CR	AVE	Cronbach Alpha	rho_A	CR	AVE
FAC	0,806	0,829	0,883	0,715	0,785	0,786	0,903	0,823
PPA	0,834	0,843	0,901	0,754	0,762	0,765	0,894	0,808
SE	0,883	0,885	0,928	0,811	0,819	0,843	0,892	0,734
SM	0,875	0,880	0,923	0,801	0,836	0,836	0,924	0,859

Own source

Fornell and Bookste in [105] analysis was performed to analyse discriminant validity. The amount of variance between its indicators (AVE) was examined, which must be greater than this variable’s variance with others in the model [110]. A second more rigorous analysis is called heterotrait-monotrait (HTMT), whose value must be < 0.90 [111] (see Table 6). In our case, the criterion is met.

**Table 6:** Discriminant validity Hotel Managers

	Fornell-Larcker Criteria				Heterotrait-Monotrait Ratio (HTMT)				
	HP	HT	PRINT	S	HP	HT	PRINT		S
HP	0.870	0.871	0.870	0.770					
HT	0.884	0.889	0.886	0.795	0,759				
PRT	0.848	0.849	0.848	0.736	0,779	0,789			
S	0.907	0.909	0.908	0.766	0,665	0,770	0,781		

Tourists

The model should be analysed using the value obtained from the root mean square residual (SRMR). In our case, the value was 0.042, which did not exceed the approved 0.08 [112].

### Analysis of internal or structural models

After ensuring that the relationships between the constructs and indicators were acceptable, the structural or internal model was evaluated by examining the relationships between the constructs to predict the model’s viability [113]. The most important value is the coefficient of determination which measures the explanatory power of the dependent variable. It can be weak, moderate, or substantial, depending on the values obtained (0.19, 0.33 and 0.67, respectively; [99]. As shown in Table 7, the R2 value is 0.553, which shows that the model is significant and adequately explains the elements that contribute to improving the happiness of bank employees. To measure the path coefficients, we used two non-parametric tests in Table 7: t-values and values. Both indicate whether the hypotheses were significant. In this case, the student’s t-value must exceed 1.96 [111], and the p-value must be less than 0.05 [114]. Both criteria are met for all hypotheses. The path coefficients (β) and t-distribution explain the significance of the hypotheses. This method is called bootstrapping and provides the model fit [111]. The confidence intervals and t-values describe a second test to assess the significance of the path coefficient. The measure is based on analysing each interval, which cannot contain a 0 value [114] (see Table 7).

**Table 7:** Results of the structural model

	Hotel Managers					Tourists				
	β	Lower CI	Higher	t Statistic	p-value	β	Lower CI	Higher	t Statistic	p-value
**H1: SHT**	0,666	0,615	0,723	22,568	**0,000**	0,681	0,685	0,043	15,829	**0,000**
**H2: SHP**	0,302	0,195	0,394	5,962	**0,000**	0,363	0,368	0,074	4,900	**0,000**
**H3: S PRT**	0,324	0,254	0,409	8,080	**0,000**	0,088	0,086	0,098	0,899	**0,369**
**H4: HTPRT**	0,258	0,130	0,373	4,035	**0,000**	0,373	0,375	0,077	4,835	**0,000**
**H5: HT HP**	0,435	0,324	0,550	7,463	**0,000**	0,473	0,466	0,073	6,493	**0,000**
**H6: HP PRT**	0,325	0,224	0,423	6,262	**0,000**	0,387	0,387	0,067	5,802	**0,000**

Own source

Table 8 also measures the predictive relevance of the second trimester by predicting endogenous latent constructs. The resulting parameter must be greater than Q2 > 0. In our case, Q2 > 0.438, this criterion is thus validated (Table 8). Path coefficients and parameters [111]

**Table 8:** Coefficient of determination (R^2^) and Stone-Geisser test (Q²)

	Hotel Manager		Rural touristic	
	Q2	R2	Q2	R2
**HP**	0,303	0,455	0,471	0,589
**HT**	0,331	0,443	0,368	0,464
**PRT**	0,498	0,620	0,426	0,595

Own source

## Discussion

According to the hypotheses obtained, we establish a difference between the response of hotel managers and tourists who have stayed in such hotels. The model meets all the hypotheses for hotel managers. Hypotheses 1 and 3 have a strong relationship between latent variables. In H1 safety seeking in rural tourism destinations and health-seeking in rural tourism destinations are strongly related (H1: S PRT: β = 0.666; T = 22,568; p-value = 0.000). According to WHO [41]safety not only is related to the service provider and their customers [1] but to the fulfillment of “safety” conditions to provide health-seeking conditions.

According to Castañeda-García, et al., [25] health-seeking conditions not only come through following the health international guidelines about vaccination but when each individual raises responsible awareness about fulfilling the Non-Pharmaceutical Interventions (NPIs) guidelines Responsible awareness goes beyond hygiene measures and actions to deter the spread of the virus. It is when the study of psychological factors plays a key role in connecting safety with health conditions within Covid-19 pandemic context [27]. Health psychological factors such as perceived susceptibility, severity, and benefit need to be studied to prevent behaviour against the risks of the spread of the virus.

According to Wang et al., [29], it is important to increase the safety awareness of individuals by the perceived severity and susceptibility to exhibit self-protective travel behaviour. On the other hand, attitudes toward the virus can release negative emotions that should be channelled toward positive personal attitudes and psychological resilience against fear [28]. The increase of health awareness to avoid reckless attitudes in relation to the virus ends up in rational tourists who tend to be risk-averse and avoid dangerous situations. According to Mitchell et al., [32] health awareness in individuals depends on risk tolerance levels. Risk avoidance should be introduced to minimise the impact of the virus by modifying the consumption behaviour and Information search for tourists. Other theories such as the optimism-pessimism theory are well connected with H1 in the sense of relating the risk to the product and feeling safer towards its consumption.

H1 also gives evidence to Protection Motivation Theory (PMT) [36]. As pandemic affects intrapersonal factors through threat-appraisal or coping-appraisal. Tourist can adapt or maladapt their behaviour to increase the health protective behaviours and the risk of avoiding it [37].

Hypothesis 3: The search for safety in the rural tourism destination (S) influences the promotion of the rural tourism destination (PDT) (H3: S PRT: β = 0.324; T = 0.808; p-value = 0.000). H3 points to the important role that health authorities play in ensuring safety conditions in tourist destinations in the new Covid-19 era. The promotion of destinations stems from the consequences of the terrible effects of the pandemic on the tourism industry [18]. These results have generated a paradigm shift in the tourism industry [115].In this new situation, tourism destinations have to adapt not only to the new security conditions but also to a possible contagion alert that could affect the reputation of destinations.

The promotion of destinations in the search for new opportunities and identifying new options that lead to a more responsible tourism model in terms of health factors [116].The recovery of mobility in tourism after the closure of borders and airports and the new willingness to pay tourists requires the implementation of security measures combined with new technology and digitalisation processes.

New studies in the field of tourism have shown how technology contributes to ensuring the well-being and safety of both tourists and residents [117]. The development of virtual reality products and digitalisation during the pandemic has contributed to the promotion of tourism destinations. According to the World Travel and Tourism Council (WTTC,2020) [118] the resurgence of tourism in the new post-Covid 19 era has been based on two main factors: investing in technology and proposing environmental sustainability measures.

Technological innovation in tourism addresses concerns related to the above four factors with particular emphasis on issues related to the safety of tourists’ lives, protection against contagion, etc., and health aspects during the Covid-19 pandemic. The use of technology thus becomes a suitable tool to promote destinations and the safety of visitors and residents [119].

In the case of tourists, hypothesis 3 (H3: S PRT: β = 0.088; T = 0.899; p-value = 0.369) is not significant. It means that there is no direct relationship between safety and desire to repeat in the destination for tourists. This relationship is indirect. When safety is guaranteed in the tourist destination, tourists experience happiness (H2: S H.P.:β = 0.302; T = 5.962; p-value = 0.000). This state of happiness reached through the enjoyment of a safe destination subsequently promotes the rural hotel (H3: S PRT: β = 0.387; T = 5.802; p-value = 0.000). Therefore, safety understood as an isolated phenomenon needs subsequent experience to become a tool for promoting the destination as a tourist destination.

In highlighting the influence of tourism experiences on the happiness experienced by tourists, authors such as Hunt and Harbor [60] accentuate this phenomenon in times of crisis, such as the current pandemic in which we live. According to Jyothis and Janardhanan [44, 62], security only translates into positive consequences for the destination when there is a connection between eudemonic motivation (happiness) and health. In this sense, tourism businesses are not enough to develop a planning and awareness effort to develop safe experiences [38] through social distancing and limiting people-to-people contact to reduce viral transmission [39]. There is a need to ensure the “experience of safety” is transformed into an experience of happiness. Rural areas are exposed to worse health services to meet their basic, social and healthcare needs, hence the importance of ensuring hygiene in rural hotels since the origin of the spread of the Covid-19 virus [41]. Hence, in the case of Spain, the important of following the guidelines issued by the Government to avoid infections and contagions in hotels [44].

About the effect of health on the pursuit of happiness among hotel managers (H5: HT HP: β = 0.435; T = 7.463; p-value = 0.000) and tourists (H5: HT HP: β = 0.473; T = 6.493; p-value = 0.000) in both cases, the relationship is significant. Results such as those obtained allow us to contribute to the literature to provide empirical analyses of both constructs to possess solid literature on the impact these two parameters produce on health tourism [67, 68]. As expressed in the hypotheses, both hotel managers and tourists allow accepting health tourism management models that pivot on the holistic pursuit of tourists’ happiness in each tourism destination [70].

**Fig. 2:**
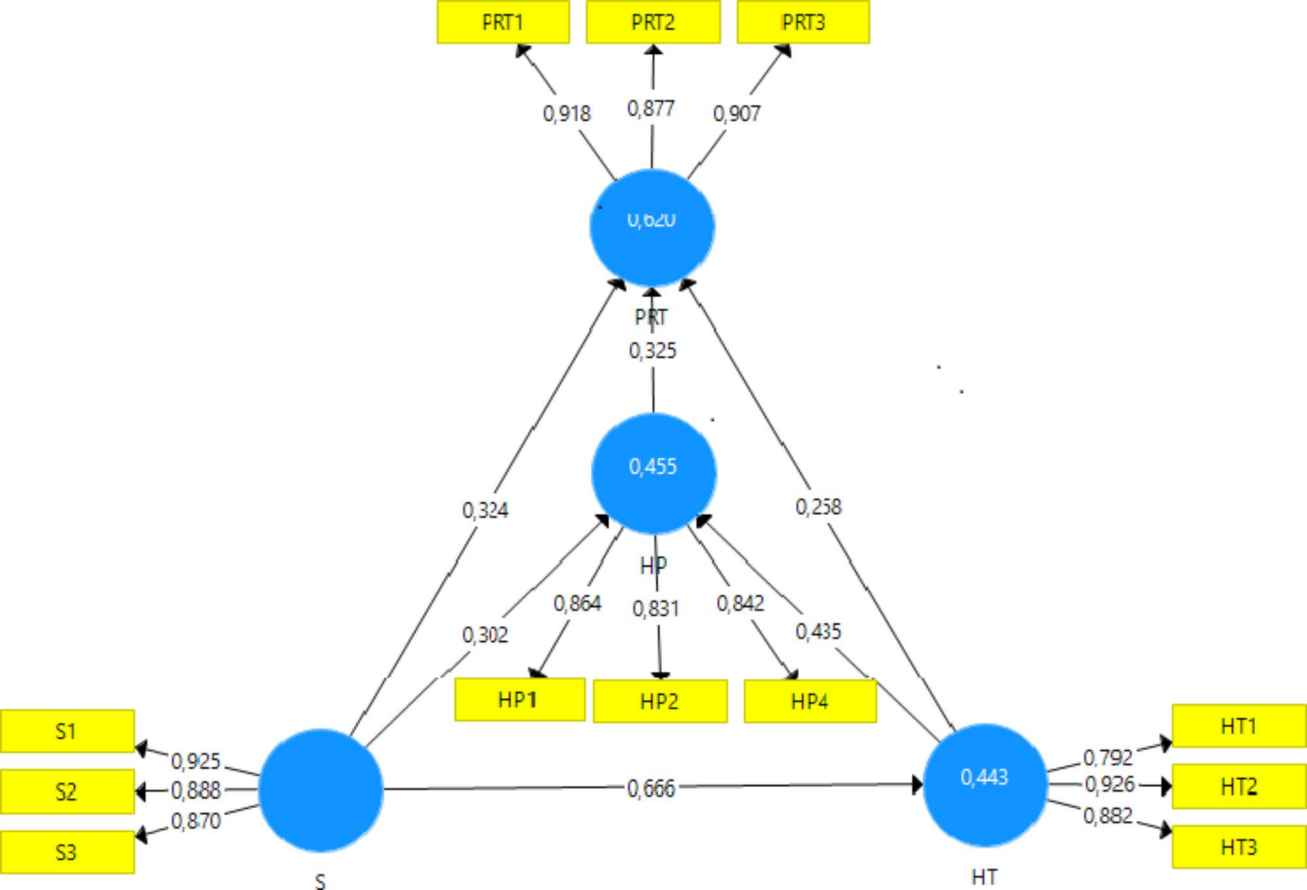
Model hypothesis and indicators

Similarly, health becomes an essential factor of tourism promotion in rural hotels for hotel managers (H4: HT PRT: β = 0.258; T = 4.035; p-value = 0.000) and the desired factor for customers of such tourism destinations (H4: HT PRT: β = 0.373; T = 4.835; p-value = 0.000). It allows tourism consumers to enjoy great positive experiences and carry healthy habits in tourist accommodations. Both aim to develop happiness [11]. Given the above, the promotion of destinations leads tourism managers to cultivate a management model based on the theories of happiness management and the search for the subjective well-being of its users through the parameters of quality, health and safety [12]. Figure 2 provides an overview of the value of each of the hypotheses and the values obtained for each of the variables and indicators.

## Conclusions

After the Covid-19 eruption, a vision and approach to tourism destinations have expanded throughout the industry. Let us analyse the effect that both safety and health have on the happiness of hotel managers and tourists in the tourism industry in Spain. We can draw several conclusions from the study conducted.

The first conclusion is that promoting tourist destinations under safe and healthy conditions has become a priority objective in the tourism industry [8]. However, while good tourism product design based on those attributes might be enough for hotel managers, it is not enough for tourists. It is necessary to connect the health and safety experience very well with the tourism product design. In this way, the tourist will experience and expectation of happiness that will promote that tourist destination [117].

The second conclusion that follows from the first is that the variables safety and health and the pursuit of happiness are essential factors in promoting tourist destinations for rural hotel managers and rural tourists [117].

The third conclusion related to the first two is that the opportunity that this study provides to develop strategies of an innovative, sustainable, and creative nature based on the relationships of the new trinomial of health, safety and happiness, from the perspective of happiness management [116]. It will enable the top managers of the rural tourism industry to drive tourism experiences through quality, happiness, and excellence in the post-Covid-19 era. In this way, hotel establishments become ecosystems that holistically promote the health and safety of their workers and their customers.

This study has had some limitations. First, the difficulty of organising joint variable selection sessions among managers of rural hotels. Therefore, the number of sessions had to be multiplied to reach the most significant number of participants in the study. It has caused a loss of information as it has not been possible to interact live among the hotel managers. The information has been processed and introduced in general terms at the beginning of each of the sessions. Secondly, it does not consider temporal and cross-sectional effects. Thirdly, that only hotel managers and tourists in Spain were surveyed. It means that the results arrived at in this research may be geographically limited. Moreover, the fourth, which has already been discussed throughout this article, is the scarcity of literature that empirically examines how the dimensions of safety, health and happiness influence the tourism promotion of rural hotels.

Among the lines of future research, the research team proposes to specify further the factors that because happiness based on the measurement of authentic experiences to build a new model of hotel tourism promotion. Detailing the quality and variety of these experiences will allow hotel managers to focus on designing and taking more realistic, healthier, safer, and happier measures in the new dimension of the pandemic.

### Theoretical implications

Since the emergence of the Covid-19 pandemic, much of the research in the tourism discipline has focused on empirically exploring the dimensions of health and hygiene safety within tourism establishments [120, 121]. The academic interest in studying these elements lies in two fundamental aspects. The first is that both dimensions are highly relevant in the decision-making process of tourists when choosing which establishment to stay in during their holidays [122, 123]. Secondly, both attributes significantly improve the satisfaction of both internal and external customers [124].

Both concepts investigate in a fragmented way in recent literature [125]. This fact may have been one of the reasons for the need for more scientific work exploring these two constructs under the happiness management approach [126]. This corporate culture stimulates guests’ positive emotions and experiences in tourist accommodations. It achieves this by implementing corporate governance to promote a positive, collaborative, and supportive work climate that proactively stimulates the happiness at work of its human capital [127]. Recognising that much remains to understand about this topic in the tourism industry, the authors of this paper believe that it may be fertile ground for research to examine how health and safety variables affect the happiness of hotel managers and tourists in the post-Covid-19 era. The results of this work show that happiness management has become a strategic resource for promoting tourist destinations.

### Practical implications

Employees in hotel establishments are characterised by carrying out their profession in an environment of high stress, anxiety, and emotional exhaustion [128]. One of the most excellent tools available to management to change this situation is to promote the happiness of their employees as a way not only to improve their standards of excellence and quality and ensure sustainable business success in the medium and long term [129].

In the context of Covid-19, the happiness at work of workers in the tourism industry becomes one of the essential instruments for corporate governance, on the one hand, to build customer loyalty in this landscape of geopolitical uncertainty. Moreover, on the other hand, to design tourism marketing campaigns that shows that their establishments are sources of psychological well-being [130].

From this perspective, the top management of tourism companies should undertake human resources policies that ensure their internal customers’ health, safety, and happiness. The empirical findings of this research show that this trinomial (health, safety, and happiness) has a very positive effect on the promotion of rural tourism destinations.

This trinomial, therefore, becomes a precious catalyst for management to develop management models that enable them to enjoy a large number of tourists. It also requires ensuring the subjective well-being of the residents of tourist destinations, who are leisure consumers [131].

Combining these two factors allows hotel establishments to become an ecosystem of positive emotions, health, and quality of life. Moreover, this will bring them significant competitive advantages and economic benefits in the post-Covid-19 era. Especially those that foster the certification of happiness management under sustainability, organisational justice, and corporate social responsibility [118].

## Data Availability

The data supporting this study´s findings are available from the corresponding author on request.
